# Decreased Expression of Semaphorin3A/Neuropilin-1 Signaling Axis in Apical Periodontitis

**DOI:** 10.1155/2017/8724503

**Published:** 2017-12-31

**Authors:** Ying Lin, Quan Xing, Wei Qin, Mary Anne Sampaio de Melo, Rui Zou, Meng Xu, Xiaolei Zhang, Hockin H. K. Xu, Zhengmei Lin

**Affiliations:** ^1^Department of Operative Dentistry and Endodontics, Guanghua School and Hospital of Stomatology & Institute of Stomatological Research, Sun Yat-sen University, Guangzhou, China; ^2^Department of Stomatology, The Third Affiliated Hospital of Sun Yat-sen University, Guangzhou, China; ^3^Zhujiang New Town Dental Clinic, Guanghua School and Hospital of Stomatology & Institute of Stomatological Research, Sun Yat-sen University, Guangzhou, China; ^4^Biomaterials and Tissue Engineering Division, Department of Endodontics, Periodontics and Prosthodontics, University of Maryland Dental School, Baltimore, MD, USA; ^5^Key Laboratory of Oral Medicine, Guangzhou Institute of Oral Disease, Stomatology Hospital of Guangzhou Medical University, Guangzhou, China; ^6^Department of Pathology, Guanghua School and Hospital of Stomatology & Institute of Stomatological Research, Sun Yat-sen University, Guangzhou, China; ^7^Center for Stem Cell Biology & Regenerative Medicine, University of Maryland School of Medicine, Baltimore, MD, USA; ^8^Department of Mechanical Engineering, University of Maryland Baltimore County, Baltimore County, MD, USA

## Abstract

Apical periodontitis (AP) is a chronic infection of endodontic origin accompanied with bone destruction around the apical region. Semaphorin3A (Sema3A) and neuropilin-1 (Nrp1) are regarded as a pair of immune regulators in bone metabolism. In this study, we firstly investigated the expression pattern of Sema3A/Nrp1 in apical periodontitis and its correlation with bone destruction. Using rat animal model, we analysed the level of mandibular bone destruction and the expression of Sema3A/Nrp1 on days 0, 7, 14, 21, 28, and 35 after pulp exposure. In addition, clinical samples from apical periodontitis patients were obtained to analyse the expression of Sema3A/Nrp1. These results indicated that the bone destruction level expanded from days 7 to 35. The number of positive cells and level of mRNA expression of Sema3A/Nrp1 were significantly decreased from days 7 to 35, with a negative correlation with bone destruction. Moreover, expression of Sema3A/Nrp1 in the AP group was reduced compared to the control group of clinical samples. In conclusion, decreased expression of Sema3A/Nrp1 was observed in periapical lesions and is potentially involved in the bone resorption of the periapical area, suggesting that Sema3A/Nrp1 may contribute to the pathological development of apical periodontitis.

## 1. Introduction

Apical periodontitis is a dental inflammatory disease caused by persistent microbial infection within the root canal system of the affected tooth and is accompanied with bone destruction around the apical region [1]. Restoration of lost periapical bone tissue is one of the major challenges in endodontic treatment [2]. Endodontic treatment is the standard of care for teeth with AP. Nevertheless, complete healing of bone or reduction in the size of apical radiolucency does not occur as expected outcomes after endodontic treatment. It has been suggested that an imbalance of the activity of osteoclast and osteoblast is one of the predominant reasons for bone loss. Previous studies have reported that the activity of osteoclasts could be suppressed by reducing cytokines involved in the initiation or progression of bone loss [3]. In addition, there are factors that help to maintain bone preservation and promote bone formation by stimulating osteoblasts [4]. However, studies have demonstrated that it is hard to acquire satisfactory results of bone protection by only inhibiting osteoclastic bone resorption or increasing osteoblastic bone formation [5–7]. In 2012, Hayashi et al. initially reported that Semaphorin3A (Sema3A), coupled with its receptor neuropilin-1 (Nrp1), exhibits a significant dual effect on osteoclasts and osteoblasts [8].

Semaphorins are a family of secreted or membrane-bound glycoproteins that have been identified as crucial molecules in neurogenesis [9], angiogenesis [10], oncogenesis [11], and immune response [12]. Sema3A belongs to one of the eight subclasses of semaphorins. The action of Sema3A requires binding to its transmembrane receptors, Nrp1 and Plexin A [13]. Recent studies have shown that Sema3A exerts an osteoprotective effect by both suppressing osteoclastic bone resorption and increasing osteoblastic bone formation [8]. Binding of Sema3A to Nrp1 inhibits receptor activator of nuclear factor-*κ*B ligand- (RANKL-) induced osteoclast differentiation by inhibiting the immunoreceptor tyrosine-based activation motif (ITAM) and RhoA signaling pathways. Moreover, Sema3A and Nrp1 binding stimulated osteoblasts via the canonical Wnt/*β*-catenin signaling pathway. These reports also suggested that an osteopenic phenotype was adopted in either Sema3A or Nrp1 deficient mice since the Sema3A/Nrp1 signaling axis was disrupted, so they are incapable of inhibiting the differentiation of osteoclasts [8]. Moreover, decreased expression of osteoblast markers, such as Runx2, was detected in Sema3A-deficient and Nrp1-deficient mice. Thus, the Sema3A/Nrp1 signaling axis is a promising new therapeutic pathway in bone resorption diseases. Furthermore, Sema3A/Nrp1 are also involved in immune response [14] and have been reported to reduce inflammation [15, 16]. However, the role of the Sema3A/Nrp1 signaling axis in inflammatory bone resorption is still not fully understood.

Accumulating evidence has encouraged investigations on the potential roles of Sema3A/Nrp1 in apical periodontitis. In this study, we hypothesized that the Sema3A/Nrp1 signaling axis would significantly contribute to bone resorption in apical periodontitis. We detected the expression patterns of Sema3A/Nrp1 at different developmental stages and their association with bone destruction in a rat model of apical periodontitis. Clinical samples from apical periodontitis patients were also detected to confirm our animal model results.

## 2. Materials and Methods

### 2.1. Induction of Apical Periodontitis in a Rat Model

Seventy-two seven-week-old male Wistar rats were randomly divided into 6 groups according to the expected duration time of disease (0, 7, 14, 21, 28, and 35 days), with each group consisting of 12 rats. Dental surgery under intraperitoneal anaesthesia of 2% pelltobarbitalum natricum (30 mg/kg) was performed on all rats. Specifically, the first molars in bilateral mandibles were drilled using a #1/8 dental round bur until the pulp was exposed [17], which was regarded as the initial day (0 days) of apical periodontitis. Twelve rats in each group were sequentially sacrificed according to the set time. Complete mandibles were detached from the rats. Half of the samples were prepared for microcomputed tomography analysis and histological assessments, while the remaining samples were stored at −80°C for quantitative real-time polymerase chain reaction analysis. This study was approved by the Animal Ethical and Welfare Committee of Sun Yat-sen University (Approval Number IACUC- DB-15-1204).

### 2.2. Collection of Periapical Lesions in Clinical Patients

The tissues of periapical lesions were obtained from patients with apical periodontitis during apical surgery (apical periodontitis group, AP group) (*n* = 6). The diagnosis of patients with apical periodontitis was based on clinical symptoms and cone beam computer tomography (CBCT) examination. Healthy control tissues were obtained from patients with the impacted third molar extraction surgery (control group) (*n* = 6). These patients revealed the impacted third molar in routine checkups by X-ray examinations but they had no pain and swelling. This study was approved by the Ethical Review Committee of Guanghua School of Stomatology, Sun Yat-sen University (Approval Number ERC-2015-12). Written informed consent was obtained from all patients involved in this study. Samples were then prepared for immunohistochemistry and qPCR analysis.

### 2.3. Microcomputed Tomography (Micro-CT) Analysis

Mandibles were fixed with 4% paraformaldehyde immediately after they were dissected from the rats. Forty-eight hours later, the samples were transferred into a cylindrical sample holder (*d* = 19 mm) for micro-CT scanning (Scanco, Bassersdorf, Switzerland). Key parameters were set at 70 kV, 114 mA, 20 *μ*m increments, and 3000-millisecond integration time. The apical lesions were recognized and labeled according to the gray value of the image in each section and then reconstructed into 3-dimensional images by image analysis software (VGStudio MAX, Heidelberg, Germany). Lesion volumes were calculated to assess the level of bone destruction of apical periodontitis [18].

### 2.4. Haematoxylin and Eosin (HE) Staining

After micro-CT scanning, the samples were decalcified with 10% ethylenediaminetetraacetic acid (EDTA) disodium salt for at least 2 months and then trimmed into 8 mm × 6 mm × 4 mm blocks for dehydration and paraffin embedding. Four-micrometer-thickness serial sections were cut in the mesiodistal direction and stained with HE [19]. Sections that presented complete root canals in first mandibular molars were selected for histological examination. The pathological assessment was performed under ×25 magnification (Zeiss, Osaka, Japan) by two independent investigators.

### 2.5. Tartrate-Resistant Acid Phosphatase (TRAP) Staining

TRAP staining was performed to estimate the number of osteoclasts in apical lesions using a TRAP kit (Sigma, St Louis, MO) according to the manufacturer's instructions [20]. Five random areas around the apical lesion were selected for pathological assessment under ×400 magnification. TRAP-positive cells, multinucleated (more than 2 nuclei), were identified as osteoclasts.

### 2.6. Immunohistochemistry

To analyse the protein expression level of Sema3A/Nrp1 and RANKL, immunohistochemistry was performed on three serial sections of each sample according to previously described methods [21, 22]. Specifically, sections were incubated at 4°C for 24 hours with primary antibodies: rabbit polyclonal antibody to Sema3A (1 : 500, Abcam, Cambridge, UK), rabbit monoclonal antibody to Nrp1 (1 : 500, Abcam, Cambridge, UK), and rabbit monoclonal antibody to RANKL (1 : 100, Servicebio, Wuhan, China). Goat anti-rabbit IgG was used as secondary antibody. Negative controls were obtained by replacing primary antibodies with phosphate-buffered saline. The pathological assessment method described for TRAP staining was also applied to immunohistochemistry.

### 2.7. Double-Staining Immunofluorescence

To determine the specific localizations of Sema3A/Nrp1, double-staining immunofluorescence was performed on the sections as previously described [21]. Specifically, we used rabbit anti-Sema3A polyclonal antibody (1 : 100, Abcam, Cambridge, UK) as the primary antibody, followed by a secondary donkey anti-rabbit immunoglobulin G antibody (green). Since there is no proper anti-Nrp1 antibody of other species that can react with human tissue, the sections were incubated with rabbit anti-Nrp1 monoclonal antibody (1 : 100, Abcam, Cambridge, UK) after adequate PBS washing. A goat anti-rabbit immunoglobulin G antibody was used as secondary antibody (red). Nuclei were stained using reagent DAPI (blue). The distributions of Sema3A/Nrp1 were determined by inversion fluorescence microscope (Zeiss, Oberkochen, Germany) under ×400 magnification.

### 2.8. Quantitative Real-Time Polymerase Chain Reaction (qPCR)

To investigate the expression pattern of Sema3A/Nrp1 in apical periodontitis at the gene level, periapical lesions from rat models, along with surrounding alveolar bone tissues, were dissected under a microscope. While the clinical samples were dissected from the surgeries [23]. Samples were then ground into powder in liquid nitrogen, and total RNA was extracted using a Minibest universal RNA extraction kit (Takara Bio, Ohtsu, Japan) according to the manufacturer's instructions. RNA was then reversed-transcribed into cDNA using a Transcriptor First Strand cDNA Synthesis Kit (Qiagen, Dusseldorf, Germany). Real-time PCR was performed using a Light Cycler 480 SYBR Green I Master kit (Qiagen, Dusseldorf, Germany). Primers used for the desired sequence are shown in Table 1. The relative mRNA expression levels of Sema3A and Nrp1 were quantified compared to GAPDH using the 2^−ΔΔCT^ method.

### 2.9. Statistical Analysis

Statistical analysis was performed using SPSS 13.0 for Windows (SPSS Inc., Chicago, IL, USA). One-way ANOVA analysis and Kruskal-Wallis analysis were used to test the differences in groups, and Bonferroni analysis was applied to test the differences between groups, followed by the Pearson and Spearman correlation test. *P* < 0.05 was considered statistically significant. The results are presented as the mean ± standard deviation.

## 3. Results

### 3.1. Rat Periapical Lesion Size at Different Stages

**Figure 1:**
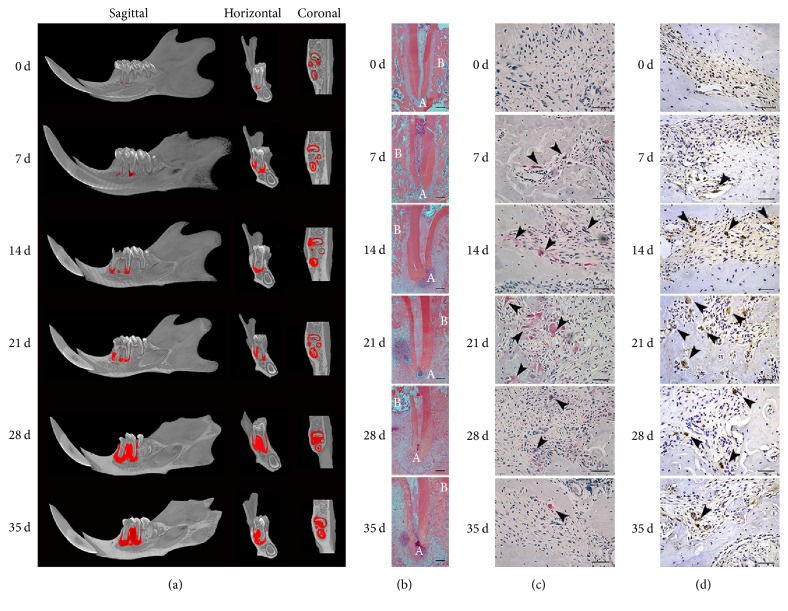
Rat periapical lesion size at different stages. (a) 3D reconstruction of a rat periapical lesion presented in sagittal, horizontal, and coronal sections after micro-CT scanning. The zone in red indicates the volume of the periapical lesion from day 0 to day 35. (b) HE staining of rat periapical lesions at different stages. Histological lesions in the sagittal section are presented under ×25 magnification, scale bar = 15 mm. (c) TRAP staining of rat periapical lesions at different stages. Osteoclasts were identified as TRAP-positive cells with multinucleated cells (arrows) under ×400 magnification, scale bar = 5 *μ*m. (d) RANKL-positive cells were stained dark brown (arrows) under ×400 magnification, scale bar = 5 *μ*m. B: bone; A: apex.

Periapical lesions were successfully induced in rats, as shown by the micro-CT results (Figures 1(a) and 3(c)) and HE staining (Figure 1(b)). The area and volume of the periapical region on day 0 were similar to those of the normal periapical space around the distal root apex of the first molar. On day 0, the periapical region was intact, and no bone resorption could be observed. According to the results of the micro-CT analysis, the lesions appeared on days 7 (marked in red), but no significant differences were detected between day 0 and day 7 (*P* > 0.05). With increased time, the volume of the apical lesions grew. The lesions continuously enlarged in the sagittal, horizontal, and coronal directions from days 14 to 35 and reached peak value on day 28. The expansion rate of the periapical lesions had slightly decreased on day 35. However, the periapical lesions on day 35 were still larger than those at other time points (*P* < 0.05) except day 28. The data are shown in Table 2.

Histological analysis by HE staining (Figure 1(b)) revealed that pulp necrosis and inflammatory cell infiltration with alveolar bone resorption gradually occurred in the rat periapical area. Few inflammatory cells were observed in the apical tissue. A slight infiltration of acute inflammatory cells with slight alveolar bone resorption was observed on day 7. Severe inflammatory cell infiltration occurred in the periapical tissues with prominent periapical bone resorption on day 14. Chronic inflammatory infiltration and continuously enlarged lesions were observed on days 21 and 28. On day 35, numerous inflammatory cells infiltrated the periapical tissue, but the area of the periapical lesions had mildly decreased. The results from the micro-CT analysis and HE staining indicated that bone destruction is a result of periapical inflammation.

In addition, osteoclasts were observed in periapical lesions using TRAP staining (Figures 1(c) and 3(c)). TRAP staining showed that few osteoclasts were observed on day 0. A gradual increase in the number of osteoclasts was observed from days 7 to 35 (*P* < 0.05). The osteoclasts peaked and showed greater cellularity on day 21. However, there were no significant differences between the numbers of osteoclasts on days 21 and 28 (*P* > 0.05). The number of osteoclasts was prominently reduced on day 35 (*P* < 0.05). Moreover, RANKL presented a similar expression pattern to osteoclasts (Figures 1(d) and 3(c)). A significant positive correlation between RANKL-positive cells and the number of osteoclasts was observed in our study (*r* = 0.805, *P* < 0.001). The data are shown in Tables 2 and 3.

### 3.2. Sema3A/Nrp1 Expression Was Decreased in Rat Periapical Lesions

**Figure 2:**
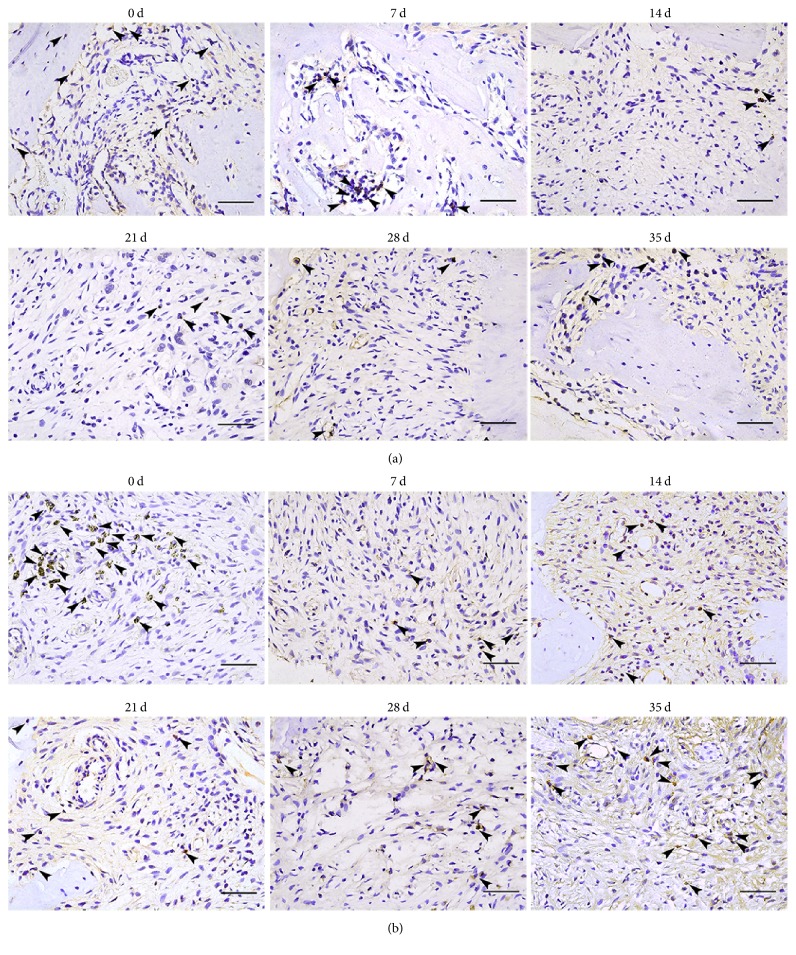
Sema3A/Nrp1 were decreased in rat periapical lesions. (a) Immunohistochemical staining of Sema3A-positive cells in rat periapical lesions at different stages. (b) Immunohistochemical staining of Nrp1-positive cells in rat periapical lesions at different stages. Cells positive for Sema3A/Nrp1 are indicated with arrows. Observation was performed under ×400 magnification, scale bar = 5 *μ*m.

**Figure 3:**
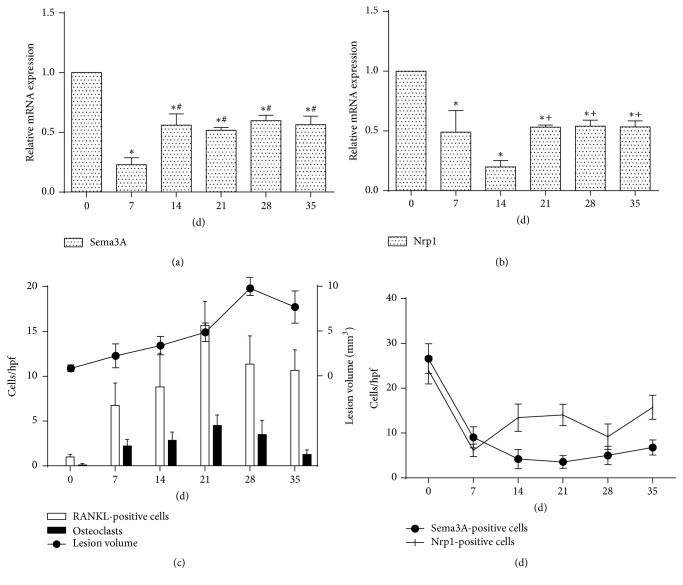
Quantitative results of expression level of Sema3A/Nrp1 and bone destruction. (a) mRNA expression of Sema3A in rat periapical lesions at different stages. (b) mRNA expression of Nrp1 in rat periapical lesions at different stages. (c) Quantitative results of RANKL-positive cells, osteoclasts, and lesion volume. (d) Quantitative results of Sema3A/Nrp1-positive cells. hpf: high power field (×400). Data are expressed as the mean ± SD (*n* = 6). ^*∗*^*P* < 0.05 versus day 0, ^#^*P* < 0.05 versus day 7, and ^+^*P* < 0.05 versus day 14.

Immunohistochemical staining analysis revealed that the number of Sema3A-positive cells was dramatically reduced from days 7 to day 35 (Figures 2(a) and 3(d)). The number of Sema3A-positive cells was significantly lower at the other time points compared with day 0 (*P* < 0.05). The lowest number of Sema3A-positive cells was on day 21. No significant difference was observed from days 14 to 35 (*P* > 0.05). The data are shown in Table 2. Furthermore, qPCR results demonstrated decreased mRNA expression of Sema3A from days 7 to 35 (Figure 3(a)). There was a significant difference in Sema3A mRNA expression between day 0 and the other time points (*P* < 0.05). The minimum detectable expression of mRNA of Sema3A was on day 7. From days 14 to 35, the expression of Sema3A mRNA was slightly increased compared to day 7 (*P* < 0.05) but was still less than that on day 0 (*P* < 0.05).

The results from the immunohistochemical studies revealed that the number of Nrp1-positive cells was dramatically decreased on day 7 compared to day 0 (Figures 2(b) and 3(d)). The number of Nrp1-positive cells was significantly lower at the other time points compared with day 0 (*P* < 0.05). Compared with day 7, the number of Nrp1-positive cells had slightly increased on days 14 and 21 (*P* < 0.05). Furthermore, there was a reduced number of Nrp1-positive cells on day 28. The number of Nrp1-positive cells slightly increased on day 35. The data are shown in Table 2. In addition, qPCR results showed that mRNA expression of Nrp1 decreased from days 7 to 35 (Figure 3(b)). There was a significant difference in Nrp1 mRNA expression between day 0 and the other time points (*P* < 0.05). The lowest expression of Nrp1 mRNA was at day 14. From days 21 to 35, the mRNA expression of Sema3A was slightly increased compared to day 14 (*P* < 0.05) but was still decreased compared to day 0 (*P* < 0.05).

### 3.3. Decreased Sema3A/Nrp1 Expression Was Correlated with Bone Destruction in Rat Periapical Lesions

The results indicated that the number of Sema3A-positive cells had a significantly negative correlation with lesion volume (*r *= −0.637, *P* < 0.001), number of osteoclasts (*r *= −0.893, *P* < 0.001), and number of RANKL-positive cells (*r *= −0.850, *P* < 0.001). With regard to Nrp1, there is a relatively weak correlation between Nrp1-positive cells and lesion volume, osteoclasts, and RANKL-positive cells. The data are shown in Table 3. Taken together, these findings suggested that decreased Sema3A/Nrp1 expression was correlated with osteoclast formation and bone destruction in rat periapical lesion.

### 3.4. Sema3A/Nrp1 Expression Was Decreased in Apical Periodontitis Patients

**Figure 4:**
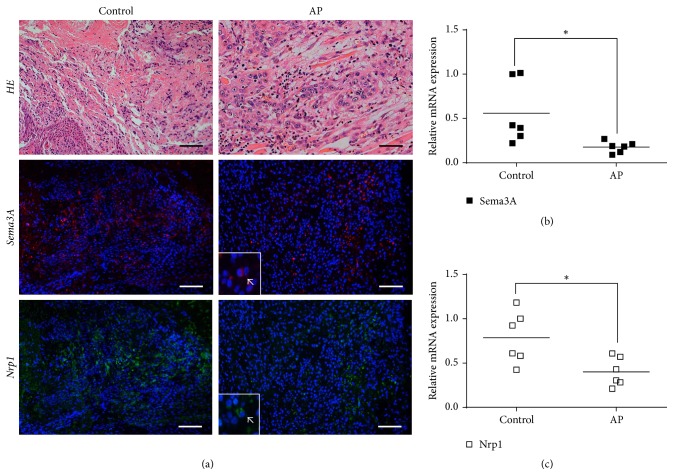
Expression pattern of Sema3A/Nrp1 in apical periodontitis patients. (a) Histological observation of periapical region in control group and AP group under ×200 magnification, scale bar = 50 *μ*m (first panel). Double-staining immunofluorescence for Sema3A/Nrp1 in apical periodontitis patients under ×400 magnification, scale bar = 25 *μ*m (second and third panels). The nuclei are stained in blue. Sema3A (red) and Nrp1 (green) are mainly expressed in the cytoplasm. (b) mRNA expression of Sema3A in the control group and the AP group. (c) mRNA expression of Nrp1 in the control group and the AP group. Data are expressed as the mean ± SD (*n* = 6). ^*∗*^*P* < 0.05 between groups.

An assessment of inflammation indicated that the samples from AP group showed more neutrophils, macrophages, lymphocytes infiltration, and angiogenesis compared to the samples from the control group (Figure 4(a), first panel). In contrast, a decreased number of Sema3A/Nrp1-positive cells were observed in the AP group compared to the control group according to the double-immunofluorescence staining (Figure 4(a),* second and third panels*). Moreover, the qPCR results demonstrated that the mRNA expression of Sema3A/Nrp1 was significantly decreased in the AP group compared to the control group (*P* < 0.05) (Figures 4(b) and 4(c)).

## 4. Discussion

Apical periodontitis is a type of disease characterized by inflammation and bone destruction. Bone loss in the apical region around the teeth results from the activity of osteoclasts and the suppression of osteoblasts—an imbalance in bone metabolism. The process of bone remodeling has been proposed to consist of three phases: bone resorption, transition, and bone formation [24]. Sema3A has been identified as a soluble molecule with the ability to exert a dual effect on both osteoblasts and osteoclasts, which results in an increase in bone minerals in the third phase of bone remodeling. It has also been reported that Sema3A suppresses osteoclastic bone resorption and can increase osteoblastic bone formation [8]. However, the role of Sema3A in inflammation-derived bone destruction is not fully understood.

In our study, the expression levels of Sema3A and Nrp1 were remarkably decreased in induced rat apical periodontitis as the disease progressed. The clinical samples obtained from apical periodontitis patients also demonstrated downregulated expression levels of Sema3A and Nrp1 in apical periodontitis compared to healthy control. Furthermore, this study revealed that Sema3A /Nrp1 expression had a significantly negative correlation with bone destruction.

Recent advances have made it increasingly clear that Sema3A predominately exerts a critical effect on bone homeostasis by inhibiting bone resorption and enhancing bone formation. Decreased Sema3A expression has been proposed to lead to a trend of decreasing effectiveness in promoting osteoblast formation in bone metastases of human breast cancer cells, suggesting that decreased Sema3A may play an important role in bone loss [11]. In addition, we found that Sema3A/Nrp1 expression exhibits a significantly negative correlation with osteoclast formation in apical periodontitis. Sema3A is generally expressed in bone tissues, but RANKL is occasionally induced by proinflammatory cytokines such as TNF-*α*, IL-1, and IL-17 [25]. Studies have shown that ITAM and RhoA signaling pathways can be activated by RANKL, resulting in resorption in bone tissues [8]. In apical periodontitis, the expression of Sema3A is decreased, along with the reduced binding of Sema3A and Nrp1. Therefore, the ITAM and RhoA signaling pathways are not effectively inhibited by Sema3A/Nrp1 signaling axis and the lesion will expand as time extends.

Thus, these findings suggested that downregulation of Sema3A/Nrp1 might lead to increased osteoclast activity. Combined with our results, it is highly likely that the reduction in Sema3A/Nrp1 expression was involved in the occurrence and developmental process of bone loss in apical periodontitis.

Many proinflammatory cytokines expressed in periapical sites are responsible for stimulating bone resorption accompanied by periapical inflammation. A recent study revealed that Sema3A expression was downregulated in rheumatoid arthritis [15] and inflammatory bowel disease [26], which was in accordance with our results. Meanwhile, studies showed that Sema3A binding to Nrp1 alleviated inflammation and the progression of autoimmune arthritis by reducing anticollagen IgG levels and suppressing the release of IFN-*γ* and IL-17 [16], which are considered to be critical in mediating inflammation in diseases [14]. Thus, we proposed that the relationship between Sema3A/Nrp1 and inflammation was interactive. Decreased Sema3A/Nrp1 expression can deteriorate inflammation and application of Sema3A/Nrp1 might reduce inflammation in the apical lesion, which needs further investigation in the future.

Sema3A administration had been proven to prevent bone loss and promote bone formation in several animal models of bone loss conditions [27]. Further* in vitro *studies are required to determine the specific role of Sema3A and Nrp1 in different types of periodontal cells. Moreover, the imbalance in Sema3A and Nrp1 may also affect the etiology of apical periodontitis. Further elucidation of the role of Sema3A/Nrp1 in this disease may address the biological and pathological functions of Sema3A/Nrp1 and allow the development of new strategies for the treatment of apical periodontitis.

## 5. Conclusions

This study demonstrated that Sema3A/Nrp1 expression was significantly decreased in rat apical periodontitis models as well as in apical periodontitis patients. The reduction in Sema3A/Nrp1 expression may have a suppressive effect on the pathological severity of apical periodontitis by inhibiting osteoblasts, activating osteoclasts, and enhancing inflammation.

## Figures and Tables

**Table 1:** Primers used for qPCR.

Species	Gene	Primer	
		Forward	Reverse
Rat	GAPDH	GGCTCTCTGCTCCTCCCTGT	CGTTCACACCGACCTTCACC
	Sema3A	CTGCTCCGACTTGCAGCATC	CGCCTCTGAAATTGCCAATATACC
	Nrp1	GAAAGGCGACAAGAACATC	TACAGCACAACTCCACAGAC

Homo	GAPDH	GAGTCCACTGGCGTCTTCAC	GTTCACACCCATGACGAACA
	Sema3A	CAGCCATGTACAACCCAGTG	ACGGTTCCAACATCTGTTCC
	Nrp1	CGCTACGACCGGCTAGAAAT	AGAGAATGCCCGATGAGGAT

**Table 2:** Lesion volume and number of osteoclasts, RANKL^+^, Sema3A^+^ cells, and Nrp1^+^ cells per high-power field (×400).

	0 d	7 d	14 d	21 d	28 d	35 d
Lesion volume/mm^3^	0.83 ± 0.36	2.23 ± 1.34^def^	3.37 ± 1.02^aef^	4.85 ± 1.03^abef^	9.75 ± 0.79^abcdf^	7.67 ± 1.79^abcde^
Osteoclasts/hpf	0.10 ± 0.17	2.23 ± 0.72^ade^	2.87 ± 0.90^adf^	4.53 ± 1.16^abcf^	3.50 ± 1.55^abf^	1.27 ± 0.53^acde^
RANKL^+^ cells/hpf	1.00 ± 0.28	6.37 ± 2.50^ad^	8.80 ± 3.80^ad^	15.68 ± 2.66^abcf^	11.37 ± 3.15^a^	10.67 ± 2.28^ad^
Sema3A^+^ cells/hpf	26.63 ± 3.31	9.07 ± 2.34^acd^	4.20 ± 2.16^ab^	3.57 ± 1.42^ab^	5.03 ± 2.06^ab^	6.80 ± 1.68^a^
Nrp1^+^ cells/hpf	24.07 ± 3.09	6.17 ± 1.37^acde^	13.43 ± 3.06^ab^	14.07 ± 2.38^abe^	9.20 ± 2.83^adf^	15.77 ± 2.68^abe^

^a^ *P* < 0.05 versus day 0, ^b^*P* < 0.05 versus day 7, and ^c^*P* < 0.05 versus day 14; ^d^*P* < 0.05 versus day 21, ^e^*P* < 0.05 versus day 28, and ^f^*P* < 0.05 versus day 35. Data are expressed as the mean ± SD (*n* = 6).

**Table 3:** Correlation analysis.

	Sema3A^+^ cells	Nrp1^+^ cells	RANKL^+^ cells	Lesion volume	Osteoclasts
Sema3A^+^ cells	Blank	*r* = 0.730^*∗∗*^	*r* = −0.850^*∗∗*^	*r* = −0.637^*∗∗*^	*r* = −0.893^*∗∗*^
Nrp1^+^ cells	Blank	Blank	*r* = −0.393^*∗∗*^	*r* = −0.347^*∗*^	*r* = −0.613^*∗∗*^
RANKL^+^ cells	Blank	Blank	Blank	*r* = 0.770^*∗∗*^	*r* = 0.805^*∗∗*^
Lesion volume	Blank	Blank	Blank	Blank	*r* = 0.519^*∗∗*^
Osteoclasts	Blank	Blank	Blank	Blank	Blank

^*∗*^ *P* < 0.05; ^*∗∗*^*P* < 0.001.
